# Thermal Degradation and Product Analysis of 3-iodo-2-propyl-butylcarbamate as a Wood Preservative

**DOI:** 10.3390/polym14214531

**Published:** 2022-10-26

**Authors:** Liping Han, Mingliang Jiang, Jingpeng Zhang, Chuang Shao, Qingshuo Zhang

**Affiliations:** 1 Research Institute of Wood Industry, Chinese Academy of Forestry, Beijing 100091, China; 2 College of Materials Science and Technology, Beijing Forestry University, Beijing 100083, China

**Keywords:** 3-iodo-2-propyl-butylcarbamate, thermal degradation, kinetics analysis, degradation products

## Abstract

The thermal degradation kinetics and degradation products of IPBC during the heating process are investigated herein. Experiments were conducted at isothermal conditions from 60 °C to 150 °C. The remaining IPBC content was analyzed by high-performance liquid chromatography (HPLC) at specific time intervals for each test, and the kinetic model of IPBC thermal degradation was established. The thermal degradation products of IPBC were studied by ultra-performance liquid chromatography-mass spectrometry (UPLC−MS/MS). The results showed that thermal degradation of IPBC occurred at 70 °C, and the degradation rate increased significantly from 70 °C to 150 °C. The thermal degradation kinetics of IPBC conformed to the first-order reaction and $k=3.47\times 10^{12}e^{-111125/RT}$ from 60 °C to 150 °C. Seven degradation products such as prop-2-yn-1-yl ethylcarbamate and methyl N-butylcarbamate were identified and the degradation reaction pathway and the mechanism of IPBC were proposed, which involved deiodination, demethylation, deethynylation, deethylation, and hydroxylation processes.

## 1. Introduction

Wood and bamboo have the advantages of high strength, sustainable utilization, and biodegradability like other natural materials. These have been used to produce wood-bamboo panels and wood/bamboo-based composites [1,2]. In the field of construction and decoration, the corresponding main products are plywood, particleboard, glulam, wood-plastic, and bamboo-plastic composite materials [3,4,5]. The rich starch contained in wood and bamboo provides the necessary nutrients for the growth and reproduction of mold and stain fungi [6,7]. Mold and blue stain fungi may reduce the quality of wood and bamboo products, especially in exterior applications. In addition, the presence of mold spores in the air may cause allergic reactions, asthma or infections [8,9,10]. The addition of anti-mildew agents in the production of these products is a common treatment method, which can effectively prevent the infection of these products by mold and stain fungi, thereby extending their service life [11].

**Figure 1:**
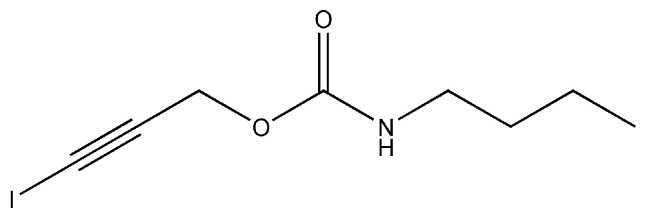
Molecular structure of IPBC.

3-iodo-2-propyl-butylcarbamate (IPBC, C_8_H_12_INO_2_, MW: 281.09) is a halogenated unsaturated carbamate broad-spectrum fungicide that consists of an iodo propynyl group and a carbamate group (Figure 1). IPBC is a white or slightly yellow crystalline powder, with a melting point of 65−67 °C, insoluble in water, and soluble in ethanol and aromatic solvents [12]. It possesses a strong inhibitory effect on various fungi and algae, such as mold, stain fungi, and yeast [12,13]. The antifungal mechanism of IPBC is still unclear, but it is speculated that it may be related to the iodo group at the end of the molecular chain. Iodine can penetrate the cell wall of microorganisms and reacts with sulfhydryl groups, which are located in nucleic acids and amino acids at the enzyme-active site of microorganisms. The active protein of the microorganism is destroyed, which can lead to the decline or loss of activity [14,15]. Since the 1980s, IPBC has been widely used in the protection of wood, bamboo and composite materials for mold and stain fungi control [12,16].

However, IPBC is a thermally unstable compound that may be decomposed under high-temperature conditions [17,18]. Thermal processing such as wood drying or hot pressing of board during wood processing might decompose IPBC and reduce its antifungal activity [19,20]. Therefore, it is necessary to accurately determine the kinetic parameters to predict changes in the IPBC content during thermal processing. At present, there are many reports on the application of IPBC as a mildew agent in wood [16,21,22,23] but few focus on the influence of thermal processing for the content and properties of IPBC. Although the thermal instability of IPBC has been reported, the specific thermal degradation kinetics and degradation mechanisms are not clear. The thermal conductivity of the material will affect the transfer of heat [24]. The thermal conductivity of wood may also affect the degradation of IPBC in the wood.

In order to ensure its efficacy for mold and stain fungi control, this paper aims to determine specific decomposition temperature and rate, decomposition kinetics, quantify the relationship between IPBC content and thermal temperature and duration, explore the degradation rule of IPBC during thermal processing, and investigate the thermal degradation of IPBC in wood samples. At the same time, the decomposition compounds of IPBC were preliminarily clarified. Overall, the results can provide a theoretical research basis for the changes in IPBC content and its characteristics during thermal processing.

## 2. Material and Methods

### 2.1. Materials

IPBC (purity ≥ 99%,) was purchased from Shanghai Shengnong pesticide Co., Ltd., Shanghai, China. Acetonitrile was obtained from Thermo Fisher Scientific Co., Ltd, Shanghai, China. The 28 years old rubber sapwood samples were from Hainan Province, China. The air-dry density and moisture content of the samples were about 0.70 ± 0.03 g/cm^3^ and 8.2 ± 0.2%, respectively.

### 2.2. Test Method

#### 2.2.1. Thermal Treatment of IPBC

The thermal treatment of the IPBC samples was conducted by first adding 0.0500 g of the powder into a conical flask with a high-temperature-resistant grinding mouth. The bottle mouth was then closed with the bottle cap and immersed in an oil bath (DF-101S, LICHEN Co., Ltd., Shanghai, China). The thermal treatment was conducted in constant temperature conditions, and the samples were tested in two temperature ranges at intervals of 10 °C: low temperature (60−100 °C) and high temperature (110−150 °C). During the heating treatment tests, three conical flasks (duplicate samples) were drawn at predetermined time intervals and placed in an ice-water bath for cooling. Then, 10 mL of acetonitrile was added to dissolve the samples for further analysis. Aluminum foil was used for shading the flask throughout the tests to avoid the influence of light during this experiment.

#### 2.2.2. Determination of the Concentration of IPBC Solution

The concentrations of IPBC were analyzed according to the reference explored previously [25]. The concentration of each solution was determined by HPLC (EliteP230, Dalian Elite Analytical Instruments Co., Ltd., Liaoning, China) with an Allsphere C18 chromatographic column (250 × 4.6 mm, 5 µm) and an ultraviolet detector. The column temperature was maintained at 30 °C and the detection wavelength was set at 200 nm. The mobile phase was acetonitrile (A) and distilled water (B) (A:B = 65:45, *v*/*v*) and was delivered at a total flow rate of 1 mL·min^−1^ with an injection volume of 20 µL.

#### 2.2.3. TG Analysis

Thermogravimetric analysis (TG) was conducted by a thermal analyzer (STA449F3 Jupiter^®^, NETZSCH, SELB, Bavaria, Germany). The temperature ranged from 30 °C to 600 °C with a heating rate of 10 °C/min. The flow rates of the purge gas of nitrogen and the air were 30 mL/min and 100 mL/min, respectively. In order to eliminate the influence of crucible thermal behavior during the experiment, all the crucibles were calcinated at 1000 °C before being used.

#### 2.2.4. Kinetic Modeling

There have been many studies on the degradation kinetics of organic compounds during thermal processing in food science. The degradation kinetic in the thermal process is generally in line with a zero-order or first-order kinetic reaction model [26,27]. Therefore, zero-order and first-order kinetic formulae were used to describe the thermal degradation of IPBC in this study.

#### 2.2.5. Thermal Treatment of Wood Samples

Rubber sapwood was cut into samples of size 20 (T) × 20 (R) × 20 (L) mm and 0.1 (T) × 20 (R) × 20 (L) mm. The samples were dipped in 0.2% (*w*/*w*) IPBC ethanol solution for 15 min at vacuum at −0.09 mPa, followed for 10 min at atmospheric pressure. After excess solution was wiped off from the surfaces of the samples, they were weighed and air dried for three weeks in a dark room. Three replicates were set for each treatment. The treated wood samples were placed in an oven for thermal treatment at 140 °C for 30 min and 60 min.

#### 2.2.6. Analysis of IPBC Content in Wood Samples

After thermal treatment, each wood sample was pulverized, 0.5 g of wood powder was extracted with 10 mL of methanol for 30 min and filtered with a 0.45-degree organic filter. The filtrate was collected for analysis.

#### 2.2.7. UPLC−MS/MS Analysis

UPLC−MS/MS analysis was performed on a Thermo UltiMate 3000 UHPLC system and a Thermo Scientific LTQ Orbitrap VELOS PRO Mass Spectrometer (San Jose, CA, USA). Chromatographic separation on the system was achieved on a Waters ACQUITY UPLC BEH C18 1.8 µm, 2.1 × 100 mm column (Waters Technology, Milford, MA, USA). The injection volume was 10.0 µL, and the mobile phase was a gradient of 0.1% formic acid water (A) and acetonitrile (B) at a flow rate of 0.4 mL·min^−1^. The elution gradient was set as follows: 0–1 min, 5–5% B; 1–30 min, 5–95% B; 30–32 min, 95–95% B; 32–32.1 min, 95–5% B; and 32.1–35 min, 5–5% B. Sample detection was carried out via heat electrospray ionization (HESI) with a capillary temperature of 350 °C, a sheath gas (N_2_) flow rate of 35 arbitrary units (arb), an auxiliary gas flow rate of 10 arb, and an ion spray voltage of 4 kV. Positive or negative ionization (ESI) modes were used for the analysis. The acquisition time was 35 min and full MS scans were acquired in the range of m/z 50−1250 with a mass resolution of 30,000. The MS/MS experiments were conducted using a data−dependent scan, and Xcalibur software (Thermo Fisher Scientific, San Jose, CA, USA) was used for data collection and analysis. In the second stage, a dynamic data-dependent scan was used; the peaks were selected in the first stage for collision-induced dissociation fragmentation scanning and detected with an ion trap dynode.

## 3. Results and Discussion

### 3.1. Effect of Temperature on Degradation of IPBC

**Figure 2:**
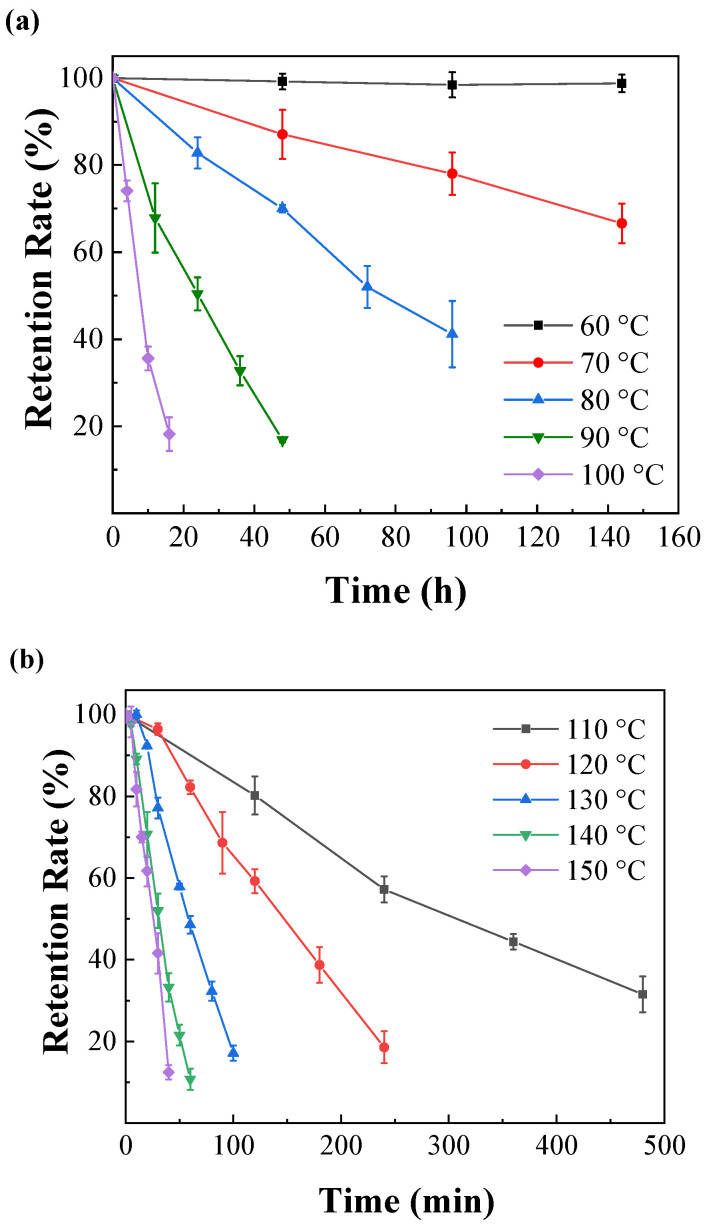
Thermal degradation of IPBC at different heating temperatures: (**a**) 60–100 °C; (**b**) 110–150 °C. Each point represents the average of three replicas per thermal treatment.

**Figure 3:**
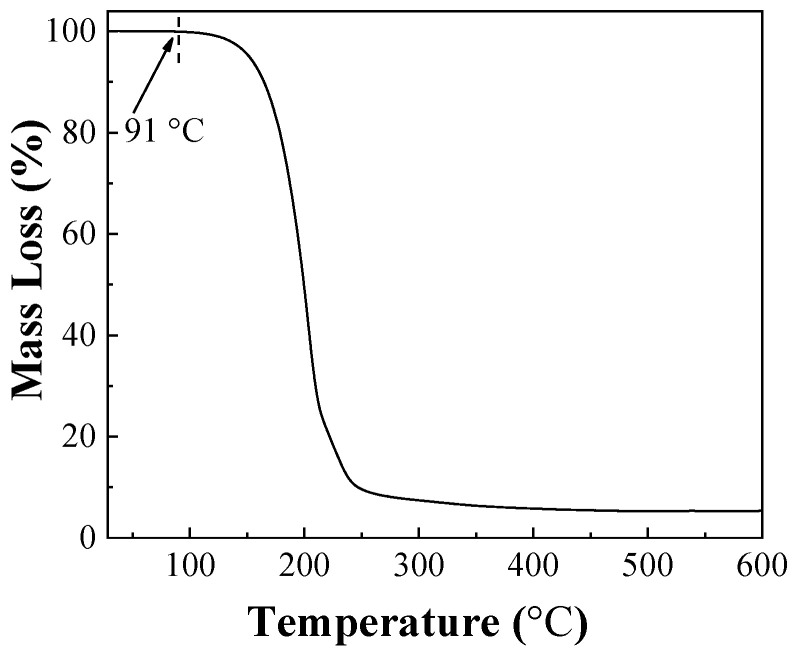
The TG curve of IPBC.

The relationship between the stability of IPBC and temperature is shown in Figure 2. IPBC was not too significantly degraded after 144 h at 60 °C, which is consistent with previous reports [28]. However, there were different degrees of degradation at 70 °C or above, which illustrated that the stability and degradation rate of IPBC has a significant correlation with heating temperature. The results of IPBC thermogravimetric test (Figure 3) showed that the starting decomposition temperature of IPBC was at 91 °C and remained stable without weight loss until 90 °C, which was consistent with previous reports [16], and also verified the thermal instability of IPBC. However, according to oil bath experiments, IPBC is also degraded to different degrees from 70 °C to 90 °C: the retention rate was still above 90% after 20 h and decreased to 65% after 144 h at 70 °C; at 80 °C, the retention rate decreased to 81.2% after 24 h and 50.9% after 72 h; and at 90 °C, the retention rate was 49.4% after 24 h and only 5.1% after 60 h. The result is different from the thermogravimetric test results, which is likely due to the bond energy of the C–I bond being 228 kJ·mol^−1^, which is lower than the bond energy of other halogens and carbon, such as the C–F (467 kJ·mol^−1^) or C–H (413 kJ·mol^−1^) bonds. At lower temperatures, IPBC undergoes the homolytic reaction [29,30]. 

**Figure 4:**
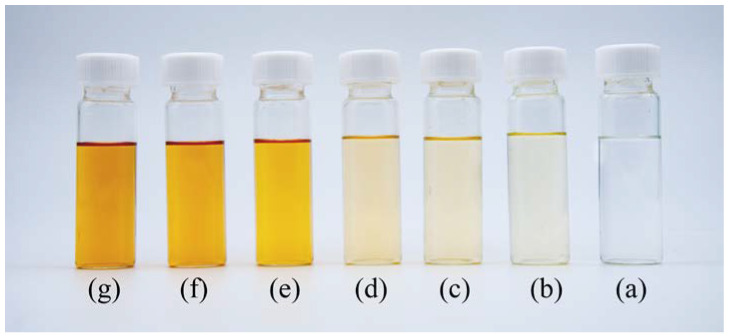
The color changes of IPBC in acetonitrile during thermal degradation at 140 °C for different heating times: (**a**) 0 min; (**b**) 10 min; (**c**) 20 min; (**d**) 30 min; (**e**) 40 min; (**f**) 50 min; and (**g**) 60 min.

The degradation rate of IPBC also increased significantly in the high-temperature range from 110 °C to 150 °C (Figure 2b), and the time required to reach the same degradation rate is significantly shorter compared with the data below 100 °C. The retention rate of IPBC dropped to 80.2% after 2 h at 110 °C, which was 10 times shorter than that at 80 °C. IPBC decreased to 59.2% after 2 h at 120 °C. The retention rate was only 5.1% after 1 h at 140 °C. The result is consistent with previous findings on the degradation of IPBC under high-temperature exposure conditions, where the presence of an iodine atom connected to a triple bond in the IPBC structure makes IPBC very labile to high-temperature-induced degradation [28]. The color of IPBC changed from white to yellow or even light brown with an increase in temperature duration during the heat treatment, which also indicated that the carbon–iodine bond had broken (Figure 4). In summary, temperature plays an important role in the degradation of IPBC. The degradation was accelerated with the increase in temperature. However, IPBC has higher stability at low temperature compared with CMIT/MIT which is completely degraded by treatment at 60 °C for 100 h [31].

### 3.2. Thermal Degradation Kinetics of IPBC 

#### 3.2.1. Determination of the Thermal Degradation Reaction Order of IPBC

The IPBC content was linearly fitted to zero-order and first-order kinetic formulae at the corresponding temperature and duration. The degradation rate constant k and the co-efficient R^2^ are shown in Table 1 and Table 2, in which k increases with temperature. Compared with the R^2^ of zero-order and first-order reactions, a suitable degradation model was determined according to the higher R^2^ value [32]. For the degradation of temperature from 60 °C to 150 °C, the corresponding sum of the coefficients for the zero-order reaction was less than the first-order reaction. 

**Figure 5:**
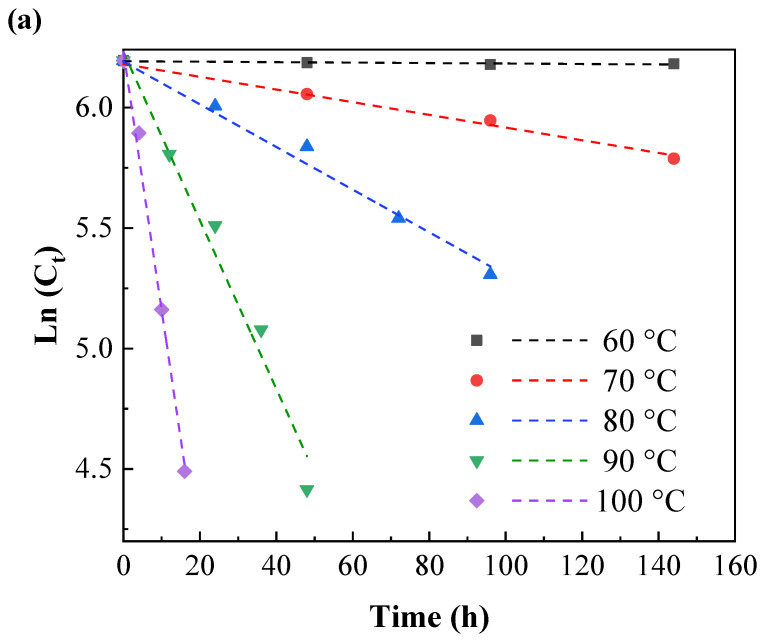

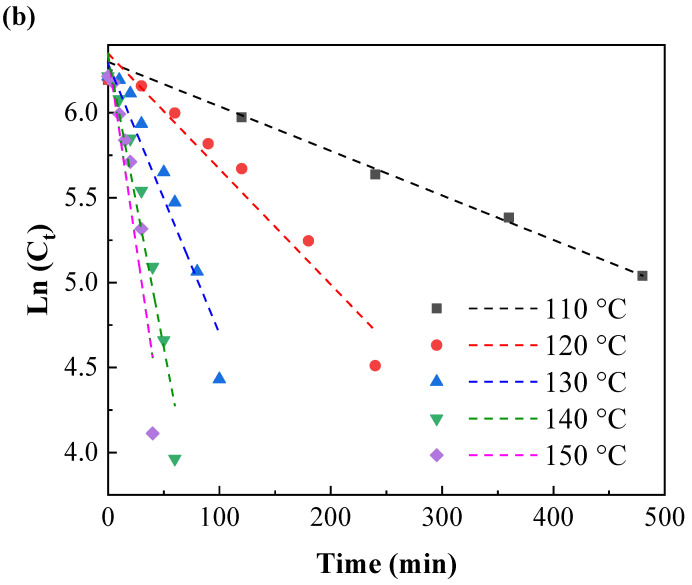
First-order kinetic model for the thermal degradation of IPBC at different heating temperatures: (**a**) 60–100 °C; (**b**) 110–150 °C.

At different temperatures, the natural logarithm of the remaining IPBC content was linearly related to the heat treatment time (Figure 5a,b). This change trend of the IPBC content was consistent with that of a first-order kinetic degradation reaction.

#### 3.2.2. Arrhenius Equation for the Thermal Degradation of IPBC

**Figure 6:**
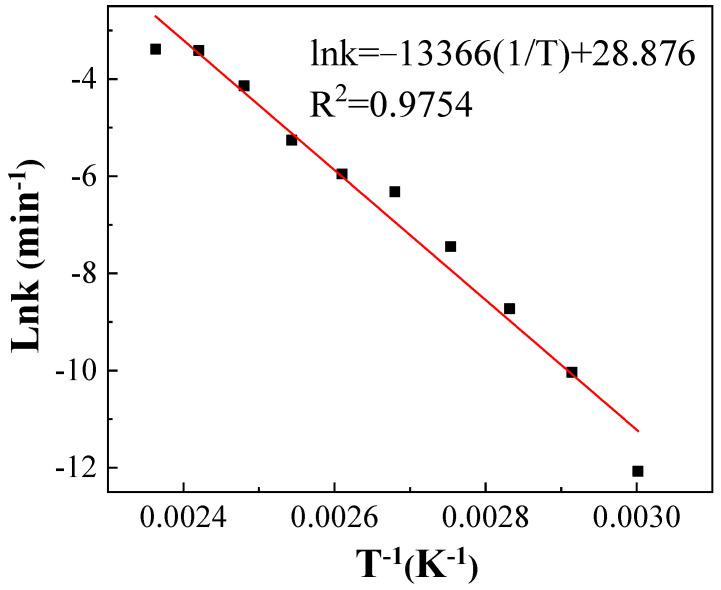
Arrhenius plot for the thermal degradation of IPBC.

Taking the natural logarithm of both sides of the Arrhenius empirical formula, the following equation is obtained:

(1) $$\ln k=\ln A-E_{a}/\mathrm{RT}$$

the natural logarithm of the first-order reaction rate constant (ln k) was linearly regressed against the reciprocal of the heat treatment temperature (1/T). The relationship between ln *k* and 1/*T* could be described as follows and shown in Figure 6:

(2) $$\ln k=-13366\left(\frac{1}{T}\right)+28.876,\;R^{2}=0.9754$$

the integral of Equation (1) provides:

(3) $$k=3.47\times 10^{12}e^{-111125/RT}$$

where R is the molar gas constant (8.314 J·mol^−1^·K^−1^) and T is the absolute temperature (K).

Equations (2) and (3) are the differential and integral formulae of the Arrhenius equation for the thermal degradation of IPBC. The activation energy Ea and the pre-exponential factor A of the reaction might be respectively obtained from the slope and intercept of the trend line. The specific parameter values are shown in Table 3.

The pre-exponential factor was 3.47 × 10^12^, and the apparent activation energy (Ea) was 111.13 kJ·mol^−1^ (Table 3). In the range of most chemical reactions (60–250 kJ·mol^−1^), the sensitivity of the thermal degradation of IPBC to temperature was moderate [33].

#### 3.2.3. Determination of the Half-Life of IPBC Degradation

The half-life of IPBC thermal degradation is only related to the reaction rate constant k and could be expressed as the following:

(4) $$t_{1/2}=0.693/k$$

The half-lives of IPBC at different temperatures are shown in Table 3. IPBC was not resistant to high temperatures, the half-life period decreases as the temperature increases, which indicated that lower temperatures help to prolong its storage period. Therefore, IPBC is suitable for storage under low-temperature conditions. Based on Equations (1) and (4), the half-life of IPBC at ambient temperature (25 °C) is 11.2 years. However, the half-life of IPBC may be shortened by biodegradation in the presence of bacteria and oxygen [29]. 

#### 3.2.4. The Application of the IPBC Thermal Degradation Kinetics in Wood/Bamboo 

There are three main methods for the IPBC treatment of wood and bamboo products: the pre-treatment of raw materials with IPBC, the addition of IPBC in wood/bamboo processing, and the post-treatment of the finished products with IPBC [34,35]. The first two methods inevitably involve the slab hot pressing process, and IPBC would need to withstand the temperature of the product material during this process [36,37]. IPBC will degrade under the above high-temperature conditions, reducing the antifungal activity [18]. Therefore, prolonged heat treatment of IPBC at higher temperature is not recommended. In the last method, the products are treated with IPBC after hot pressing and do not involve a high-temperature process. Under optional conditions, the last method should be a priority, in which a temperature less than 60 °C of kiln drying or natural air drying is suitable and recommended.

According to the thermal degradation kinetic data, IPBC will degrade above 70 °C. Therefore, the IPBC-treated wood needs to be dried under low temperature (vacuum freeze drying) and solvent ultrasonic extraction or low-temperature soxhlet extraction should be adapted for IPBC analysis in the wood.

### 3.3. Effect of Temperature on Degradation of IPBC in Wood Samples

**Figure 7:**
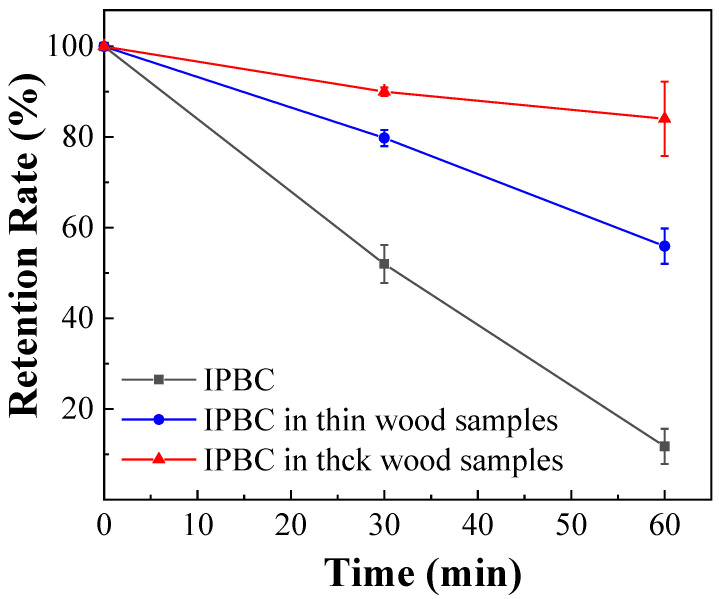
Thermal degradation of IPBC in wood samples at 140 °C.

IPBC impregnated into wood samples were also degraded by thermal treatment, which is consistent with previous studies on wood/polyvinylchloride composites (WPVC). The retention rates decreased progressively with the duration of thermal treatment as shown in Figure 7, which further verified the important effect of temperature on the stability of IPBC. However, the degradation of IPBC in wood samples was slower than IPBC (Figure 2b), which may be related to the thermal conductivity of the material [24]. Wood is a porous material, has a low thermal conductivity, and requires more time to reach the same temperature. It was found that the temperature uniformity of the oil bath is better than that of the oven, which may also cause the above experimental results during the test. Similarly, the degradation of IPBC in thick wood samples was slower than thin wood samples because the smaller the wood thickness, the faster the heat transfer. Some studies have shown that the addition of IPBC decreases the mechanical properties of WPVC due to its thermal instability [38]. At the same time, the degradation of IPBC leads to a reduction in efficacy [15]. 

### 3.4. Degradation Products of IPBC

**Figure 8:**
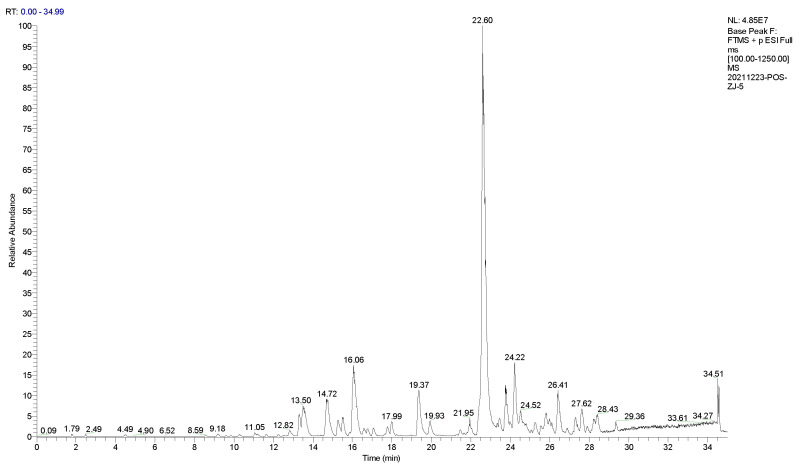
Total ion current chromatogram of IPBC degradation by UPLC−MS/MS.

**Figure 9:**
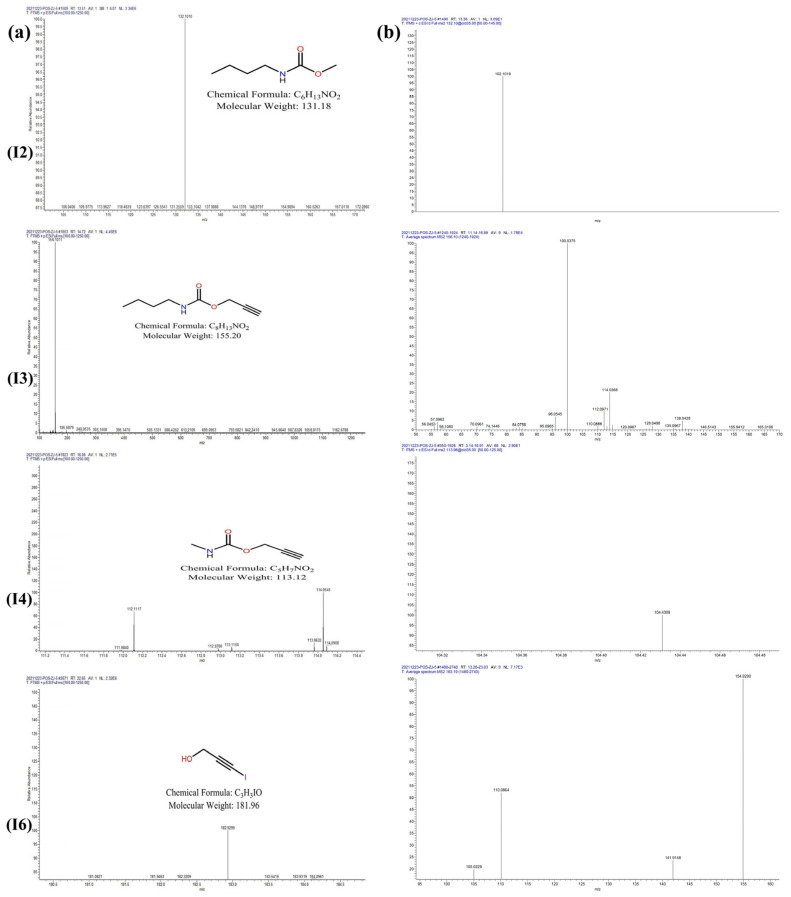
Mass spectrum of degradation products I2, I3, I4, and I6 of IPBC degradation: MS, (**a**); MS/MS, (**b**).

The main thermal degradation products of IPBC were analyzed by UPLC−MS/MS. Experimental data indicated no signal ions in the negative ion mode. Therefore, the IPBC samples were detected in positive ion mode. The degradation products of IPBC were analyzed in the full scan mode (Figure 8). The first-order fragments of the product were further broken up and detected as parent ions to obtain the second-order mass spectrum (Figure 9). The compound components, secondary ion fragments, and possible structural formulas represented by the main mass spectral peaks were inferred. The estimated results of the degradation products are shown in Table 4.

**Figure 10:**
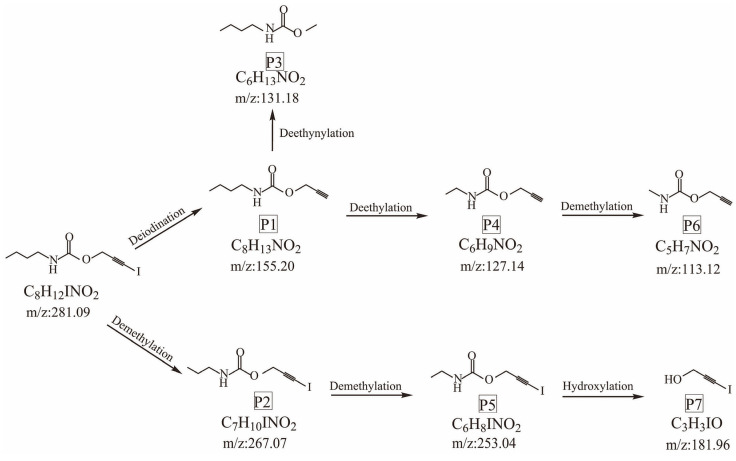
Two possible thermal degradation pathways of IPBC.

As shown in Table 4, the thermal degradation products are prop-2-yn-1-yl ethylcarbamate; methyl N-butylcarbamate; propargyl butylcarbamate; methylcarbamic acid prop-2-yn-1-ol; 2-propyn-1-ol, 3-iodo-, methylcarbamate; 3-iodoprop-2-yn-1-ol; and 3-iodoprop-2-ynyl N-propylcarbamate. Possible thermal degradation pathways for IPBC were proposed based on the mass spectrometry fragmentation data of the prototype compound and the degradation products shown in Figure 10.

There were two main pathways for the thermal degradation of IPBC. In the first degradation pathway, P1 (m/z = 155.20) was formed through deiodination, due to the relatively weak energy of the C–I bond. P1 was then converted to P3 (m/z = 131.18) by deethynylation or to P4 (m/z = 127.14) by deethylation. After demethylation, P4 was transformed into P6 (m/z = 113.12). In the other pathway, IPBC was converted to P2 (m/z = 267.02) through demethylation, and then P5 (m/z = 253.04) was formed by demethylation. Finally, P5 was transformed into P7 (m/z = 181.96) by hydroxylation. 

## 4. Conclusions

The thermal degradation at different temperatures and degradation products of IPBC were studied by HPLC and UPLC−MS/MS and the main conclusions were drawn as follow:

(1) The effect of temperature on IPBC degradation is obvious. When IPBC was heated to 70 °C, thermal degradation occurred. Therefore, IPBC should choose a low-temperature environment for transportation and storage and during the use process and is not suitable for prolonged high-temperature heating.
(2) The degradation of IPBC conformed to first-order kinetics and $k=3.47\times 10^{12}e^{-111125/RT}$ from 60 °C to 150 °C. The IPBC thermal degradation kinetic model could be used to predict the degradation loss of IPBC according to the parameters of the heat treatment process. An amount higher than the desired dosage of IPBC should be added in the production process to account for the loss of IPBC during thermal degradation to maintain the antifungal activity of IPBC in wood.
(3) The thermal degradation products of IPBC detected by UPLC−MS/MS were: prop-2-yn-1-yl ethylcarbamate; methyl N-butylcarbamate; propargyl butylcarbamate; methylcarbamic acid prop-2-yn-1-ol; 2-propyn-1-ol, 3-iodo-, methylcarbamate; 3-iodoprop-2-yn-1-ol; and 3-iodoprop-2-ynyl N-propylcarbamate. A mechanism of IPBC thermal degradation was proposed, which involved deiodination, demethylation, deethynylation, deethylation, and hydroxylation processes.

## Figures and Tables

**Table 1:** Reaction rate constants and coefficients of IPBC during thermal degradation from 60 °C to 100 °C.

Temperature (°C)	Reaction Order					
	Zero-Order			First-Order		
	k	R^2^	∑R^2^	k	R^2^	∑R^2^
60	0.0460	0.7439		5.7171 × 10^−6^	0.9999	
70	0.0186	0.9957		4.3852 × 10^−5^	0.9999	
80	0.0492	0.9959	4.6867	1.6218 × 10^−4^	0.9998	4.9992
90	0.1370	0.9770		5.8235 × 10^−4^	0.9997	
100	0.4274	0.9742		0.0018	0.9999	

**Table 2:** Reaction rate constants and coefficients of IPBC during thermal degradation from 110 °C to 150 °C.

Temperature (°C)	Reaction Order					
	Zero-Order			First-Order		
	k	R^2^	∑R^2^	k	R^2^	∑R^2^
110	0.7522	0.9865		0.0026	0.9999	
120	1.7306	0.9937		0.0068	0.9995	
130	4.4019	0.9879	4.9500	0.0159	0.9993	4.9959
140	7.9918	0.9901		0.0358	0.9991	
150	11.3843	0.9918		0.0447	0.9981	

**Table 3:** Kinetic parameters for the thermal degradation of IPBC between 60 °C and 150 °C.

Temperature (°C)	t_1/2_ (min)	Ea (kJ/mol)	A
60	121,240		
70	15,806		
80	4273.9		
90	1190.3		
100	385.1	111.13	3.47 × 10^12^
110	266.6		
120	101.9		
130	43.59		
140	19.36		
150	15.51		

**Table 4:** List of the products formed during the thermal degradation of IPBC.

No.	Retention Time (min)	Formula	Measured Molecular Mass	Theoretical Molecular Mass	Molecular Mass of Secondary Ion(s)	Compound Name
I-1	11.05	C_6_H_9_NO_2_	128.1065	127.14	111.0759	prop-2-yn-1-yl ethylcarbamate
I-2	13.50	C_6_H_13_NO_2_	132.1010	131.18	102.1019	methyl N-butylcarbamate
I-3	14.72	C_8_H_13_NO_2_	156.1011	155.20	114.0368, 100.0375	Propargyl butylcarbamate
I-4	16.06	C_5_H_7_NO_2_	114.0545	113.12	104.4309	methylcarbamic acid,prop-2-yn-1-ol
I-5	16.58	C_6_H_8_INO_2_	254.0029	253.04	235.9530, 226.0085	2-Propyn-1-ol, 3-iodo-, methylcarbamate
I-6	22.60	C_3_H_3_IO	182.9299	181.96	154.9200, 105.0229	3-iodoprop-2-yn-1-ol
I-7	23.76	C_14_H_20_I_2_N_2_O_4_	535.8060	267.07 (diploid)	418.7085, 436.6701	3-iodoprop-2-ynyl N-propylcarbamate

## Data Availability

The data presented in this study are available on request from the corresponding author.
